# Supplementary material for: Genetic and functional characterization of AMH Signaling in Zebrafish - Evidence for Roles of Amh-Bmpr2a-Bmpr1bb Pathway in Controlling Gonadal Homeostasis
Source: PLoS Genet. 2026 Mar 23;22(3):e1011958. doi: 10.1371/journal.pgen.1011958 (PMC13095126; doi:10.1371/journal.pgen.1011958)
Supplement: S1 Table — (DOCX) [file pgen.1011958.s001.docx]

**S1 Table.** Primers used for CRISPR, HRMA and qPCR

| **Gene** | **Sequence (5' to 3')** | **Application** |
| --- | --- | --- |
| *bmpr1ba* | TAGGAGCGGCTGTAGTGACGTC | CRISPR |
| *bmpr1ba* | AAACGACGTCACTACAGCCGCT | CRISPR |
| *bmpr1bb* | TAGGACGGGAAACGAAAAGTTC | CRISPR |
| *bmpr1bb* | AAACGAACTTTTCGTTTCCCGT | CRISPR |
| *bmpr1bb* | TAGGTGTGGATGGGCCGCTGG | CRISPR |
| *bmpr1bb* | AAACCCAGCGGCCCATCCACA | CRISPR |
| *amh* | AGGCAAGATTTGGGCTGATG | HRMA |
| *amh* | CTTCGGGTTGTTGTCCTGC | HRMA |
| *fshb* | AGATGAGGATGCGTGTGCTT | HRMA |
| *fshb* | GATGGAGATGTTTGTGAGTCG | HRMA |
| *lhb* | GGATGTTATTGGCTGGAAATG | HRMA |
| *lhb* | AGCGTGGAAAAACCAAGCTC | HRMA |
| *fshr* | TGTTGTCAATGATGCTGTGC | HRMA |
| *fshr* | CAGATGAAAGAGCGAGTGGA | HRMA |
| *lhcgr* | GCCCTGCTTCTTGTTTTTCTT | HRMA |
| *lhcgr* | TTACAGGTGATGCTCTTCTGG | HRMA |
| *bmpr2a* | CAGAGTGAGCAGAGGGAGTGT | HRMA |
| *bmpr2a* | CAGCGGTGTCCTTGATAACAG | HRMA |
| *bmpr2a* | GGTCTGGCCGAGCGGATTGG | HRMA |
| *bmpr2a* | GCCCCAGAGTCCAAAGCAGC | HRMA |
| *bmpr2b* | GAATCCATCCAGAAGCGGCA | HRMA |
| *bmpr2b* | GGGGTATATTTACCGGCCACA | HRMA |
| *bmpr1ba* | GTCTAGGACAGACGGCTAC | HRMA |
| *bmpr1ba* | CACTCGGCACTGAAACTC | HRMA |
| *bmpr1bb* | GGCTCCAGTCTCAGGGAT | HRMA |
| *bmpr1bb* | CGTGAGCGGACGAATAAAT | HRMA |
| *bmpr1bb* | CAGATCCAGATGGTGAAGCAGAT | HRMA |
| *bmpr1bb* | TCCTCTGTGGTGAAGAAGACCTT | HRMA |
| *amh* | TGGCGATTGGGTCGTATTGT | qPCR |
| *amh* | TCAGTGGCATGTTGGTCAGT | qPCR |
| *bmpr1bb* | GGGCGTCTTGTGGATTT | qPCR |
| *bmpr1bb* | GTGATGGTAACAGTGGCATA | qPCR |
| *bmpr1ba* | GGGACTCGTGGAGGAAAAC | qPCR |
| *bmpr1ba* | GAGTTCCCTGTGTCTCGGC | qPCR |
| *ef1a* | CCAAGGAAGTCAGCGCATAC | qPCR |
| *ef1a* | CCTCCTTGCGCTCAATCTTC | qPCR |
| *fshr* | GCTGTGCTTTATTCTTGGCTGCT | qPCR |
| *fshr* | ACCTTGTTGCCCAGACAGAC | qPCR |
| *lhcgr* | GACGGCCTGAAAGGAGTAAG | qPCR |
| *lhcgr* | GCGCAGATTCAGGTTATCAC | qPCR |
| *gdf9* | TCGTCTGATCACTCCTAGGGAA | qPCR |
| *gdf9* | GGTGGTTGTGGTCGAAGGAG | qPCR |
| *bmpr2a* | ACCGCCAGCAGTTCACTAATG | qPCR |
| *bmpr2a* | TCCGTCTTAACCAGCACATTCC | qPCR |
| *bmpr2b* | GGCTCTGCTCACTGCTTCTG | qPCR |
| *bmpr2b* | TGCGATGGCGTTGTGGTAAC | qPCR |
| *star* | ATTGAACAAGCTCTCCGGACC | qPCR |
| *star* | CCTTCTCCCCGTTGATACTCT | qPCR |
| *cyp11a1* | AGGGCCATCACCCCAATAG | qPCR |
| *cyp11a1* | CACAGCACTCACCGATTCCAG | qPCR |
| *hsd3b1* | AAGGCTCACAGGGATTTCGG | qPCR |
| *hsd3b1* | TGGCACGTTTAACCAACAGG | qPCR |
| *hsd17b1* | GCGCTCGTCTCATCTGAGTT | qPCR |
| *hsd17b1* | ACCGGCATTACACACCAGT | qPCR |
| *cyp11c1* | CTCGGGCCCATATACAGAGAG | qPCR |
| *cyp11c1* | TGTCTGCGTGTCTCTCGATG | qPCR |
| *hsd11b2* | CTGGTGGAGAGACTGTGTGC | qPCR |
| *hsd11b2* | ACAAGCCCCCACAAATCTCTTAT | qPCR |
| *cyp17a1* | GAGGCCACGGACTGTTACAA | qPCR |
| *cyp17a1* | CGATTCCCTGGCTGTACTGG | qPCR |
| *hsd17b3* | GGGAAATGGGCAGCTTTCG | qPCR |
| *hsd17b3* | AGCTTGCAGGGTATTTGGCT | qPCR |
