# Supplementary figures and images for: Genetic and functional characterization of AMH Signaling in Zebrafish - Evidence for Roles of Amh-Bmpr2a-Bmpr1bb Pathway in Controlling Gonadal Homeostasis
Source: PLoS Genet. 2026 Mar 23;22(3):e1011958. doi: 10.1371/journal.pgen.1011958 (PMC13095126; doi:10.1371/journal.pgen.1011958)

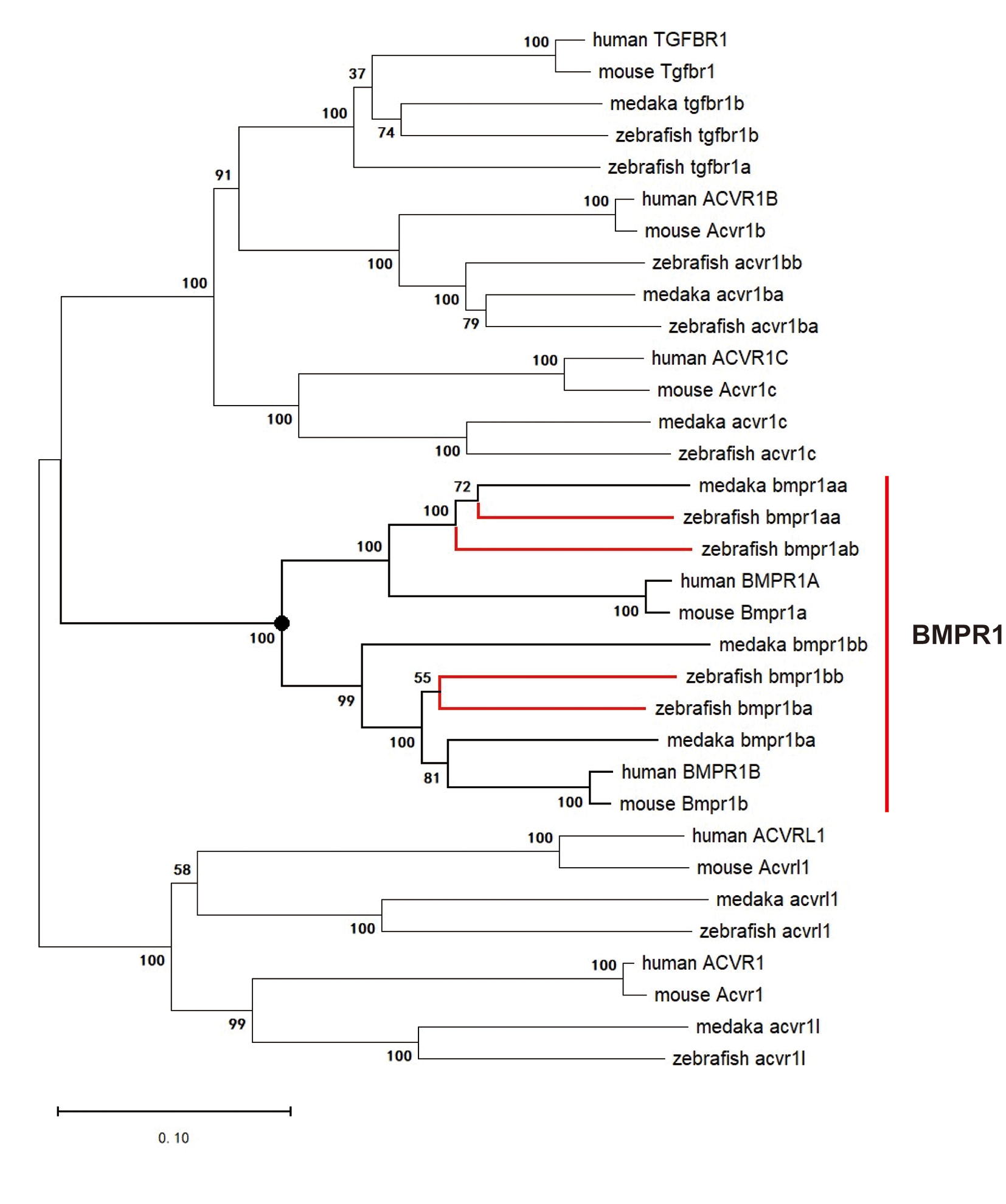

Supplement: S1 Fig — The sequences were obtained from GenBank and Ensemble databases. Sequence alignment and tree construction were performed by MEGA software using the Neighbor-Joining method. Zebrafish has two paralogous genes for both Bmpr1a (bmpr1aa and bmpr1ab) and Bmpr1b (bmpr1ba and bmpr1bb). (TIF) [file pgen.1011958.s002.tif]

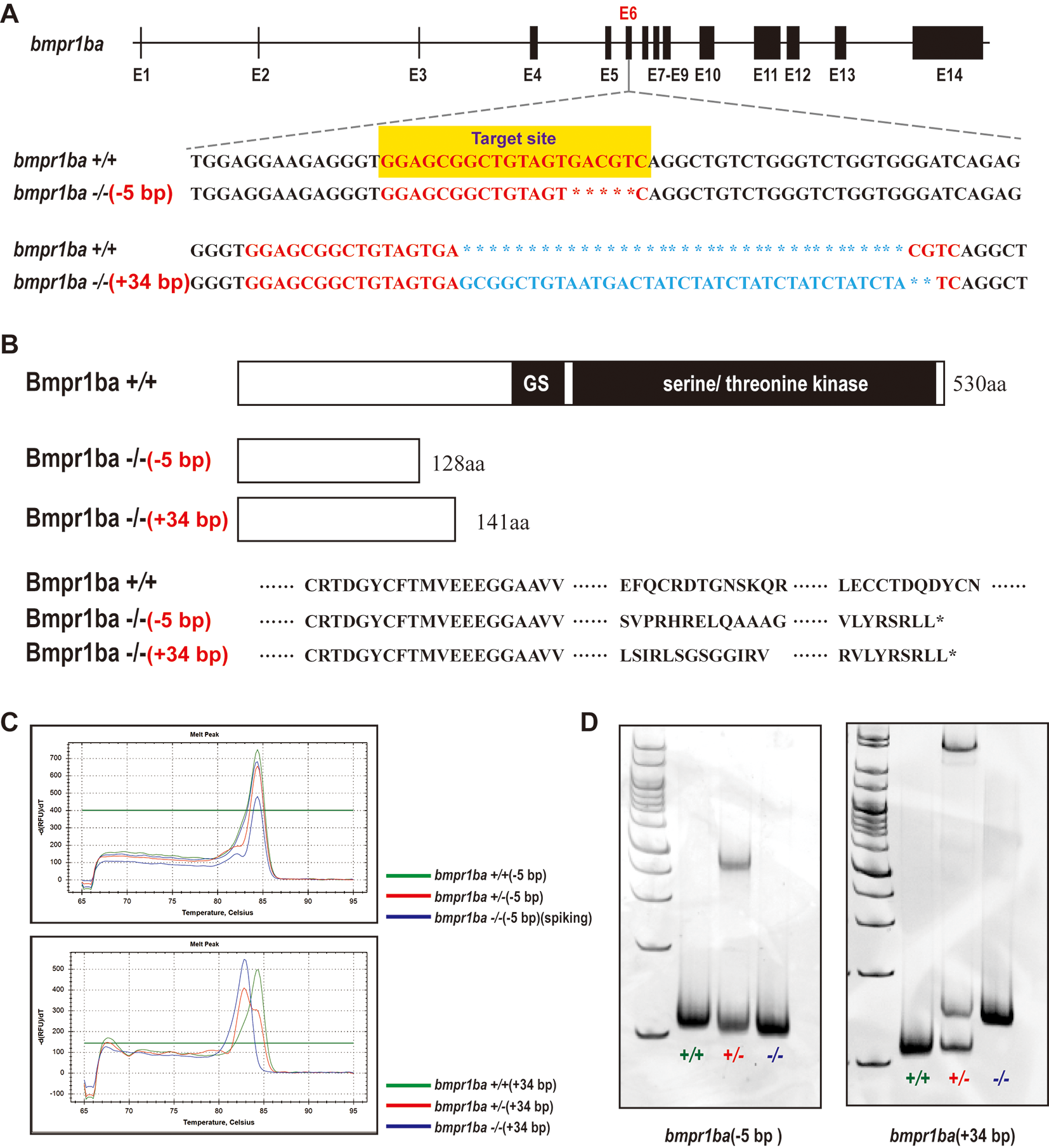

Supplement: S2 Fig — (A) Genomic Structure of the zebrafish bmpr1ba Gene. The bmpr1ba comprises 14 exons (black boxes). The CRISPR/Cas9 target site, located on exon 6, is highlighted by a yellow box. We have established two mutant lines: one with a 5-bp deletion and the other with a 34-bp insertion. (B) The schematic representation of Bmpr1ba protein sequence structure. The mutations are predicted to result in premature stop codons. (C) HRMA assay for three genotypes. (D) HMA confirmation of different genotypes of bmpr1ba mutant. (TIF) [file pgen.1011958.s003.tif]

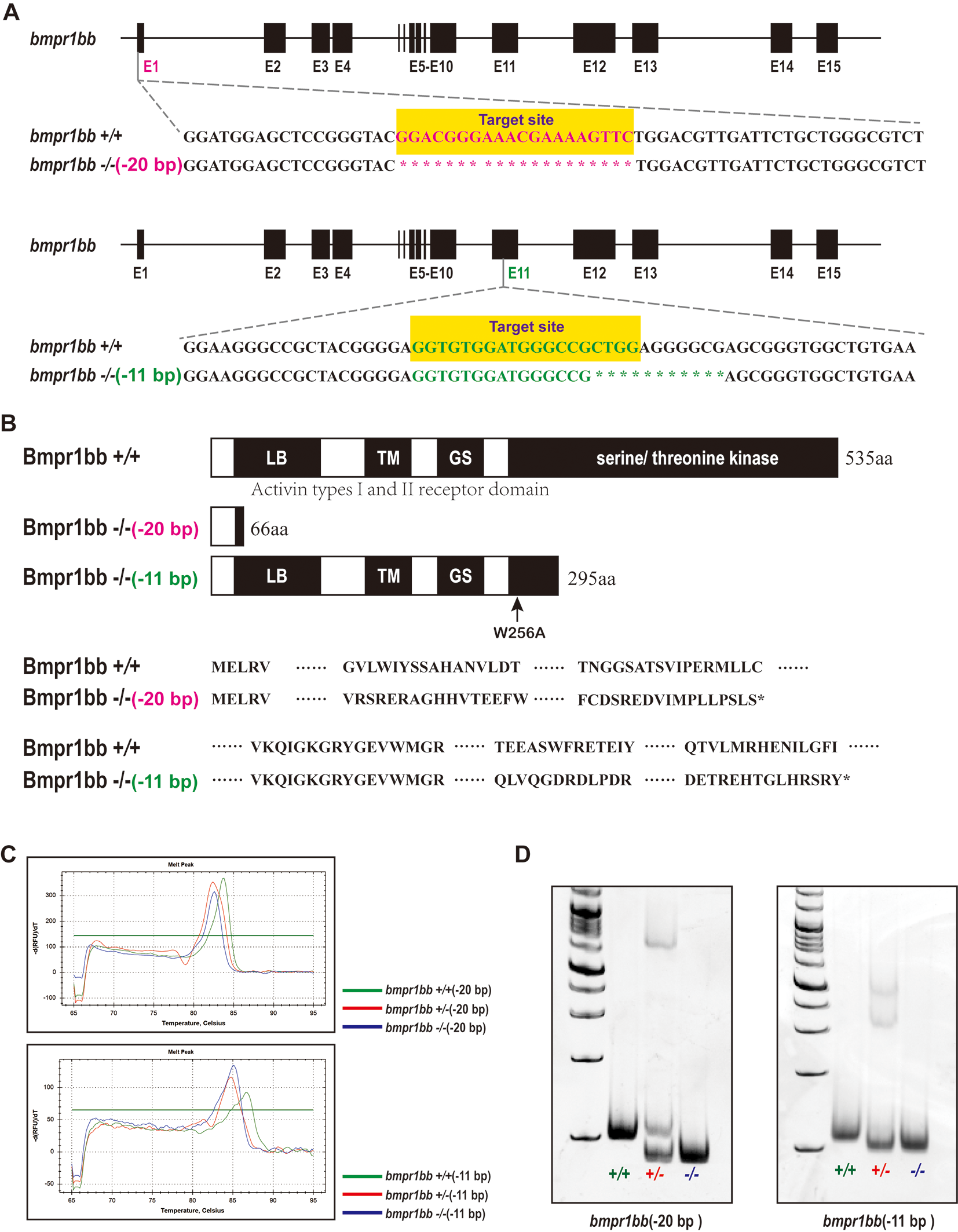

Supplement: S3 Fig — (A) Genomic Structure of the zebrafish bmpr1bb gene. The bmpr1bb gene consists of 15 exons (black boxes). Target sites for CRISPR/Cas9 editing are located in exon 1 and exon 11 (yellow boxes). Two mutant lines have been developed: one featuring a 20-bp deletion in exon 1 and another with an 11-bp deletion in exon 11. The deletions are indicated with red and green asterisks for the 20-bp and 11-bp deletions, respectively. (B) The schematic representation of Bmpr1bb protein sequence structure. The mutations result in premature stop codons. (C) HRMA assay for three genotypes. (D) HMA confirmation of different genotypes of bmpr1bb mutant. (TIF) [file pgen.1011958.s004.tif]

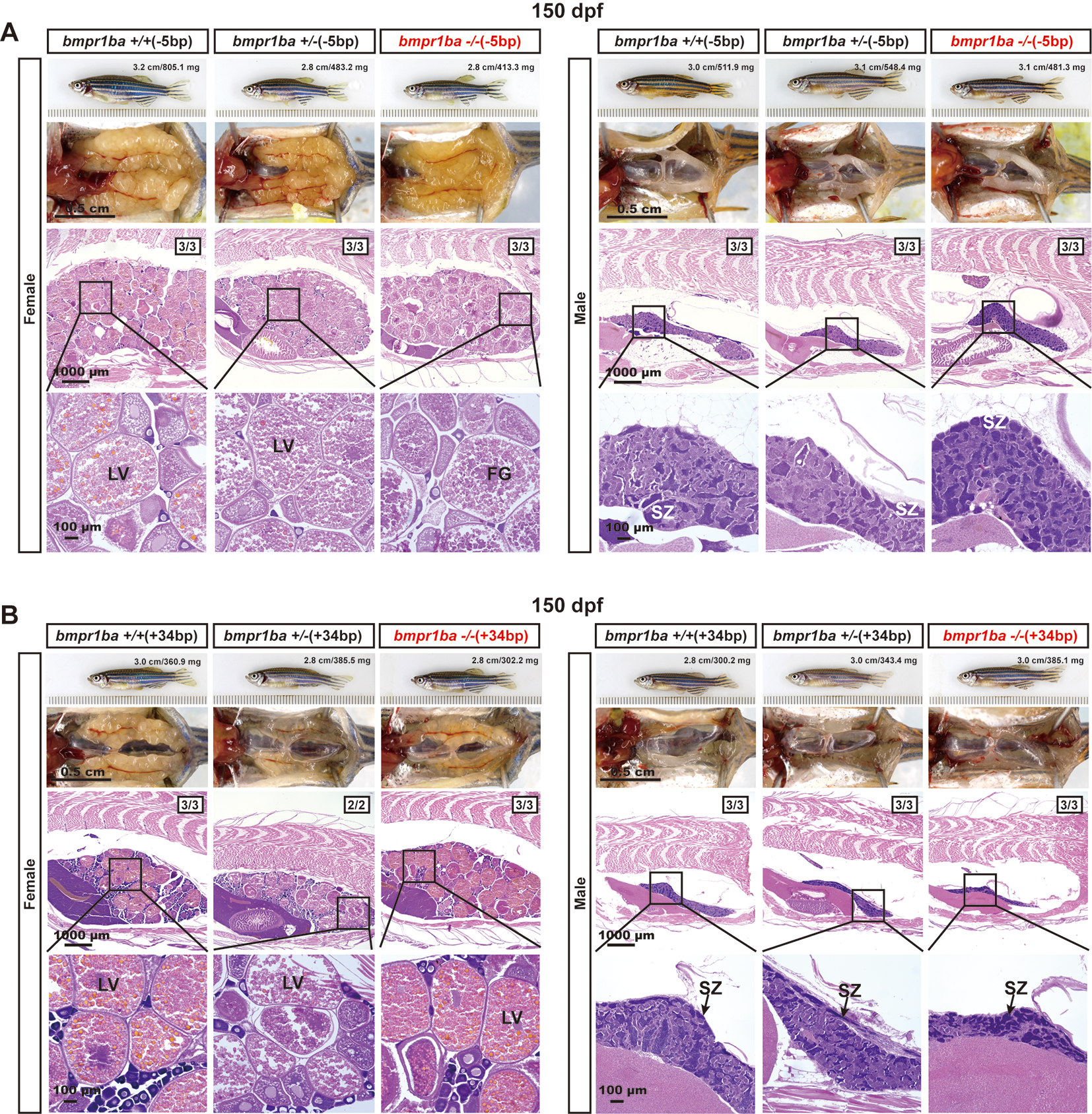

Supplement: S4 Fig — (A) Morphology and histology of bmpr1ba (-5 bp) mutant. (B) Morphology and histology of bmpr1ba (+34 bp) mutant. Both males and females showed normal gonadal growth and gametogenesis. LV, late vitellogenic; FG, full-grown; SZ, spermatozoa. (TIF) [file pgen.1011958.s005.tif]

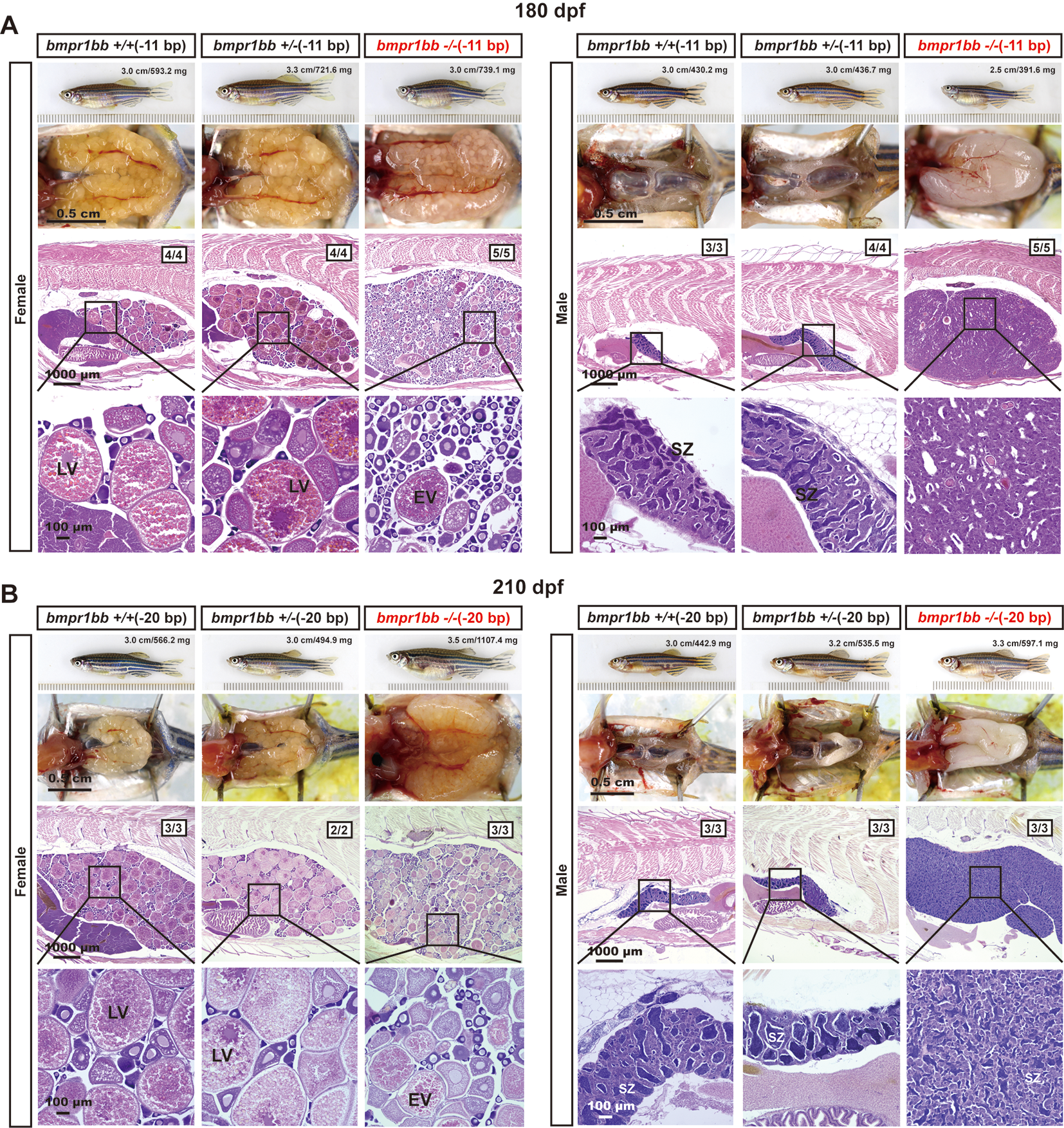

Supplement: S5 Fig — (A) Morphology and histology of bmpr1bb (-11 bp) mutant at 180 dpf. (B) Morphology and histology of bmpr1bb (-20 bp) mutant at 210 dpf. EV, early vitellogenic; LV, late vitellogenic; SZ, spermatozoa. (TIF) [file pgen.1011958.s006.tif]

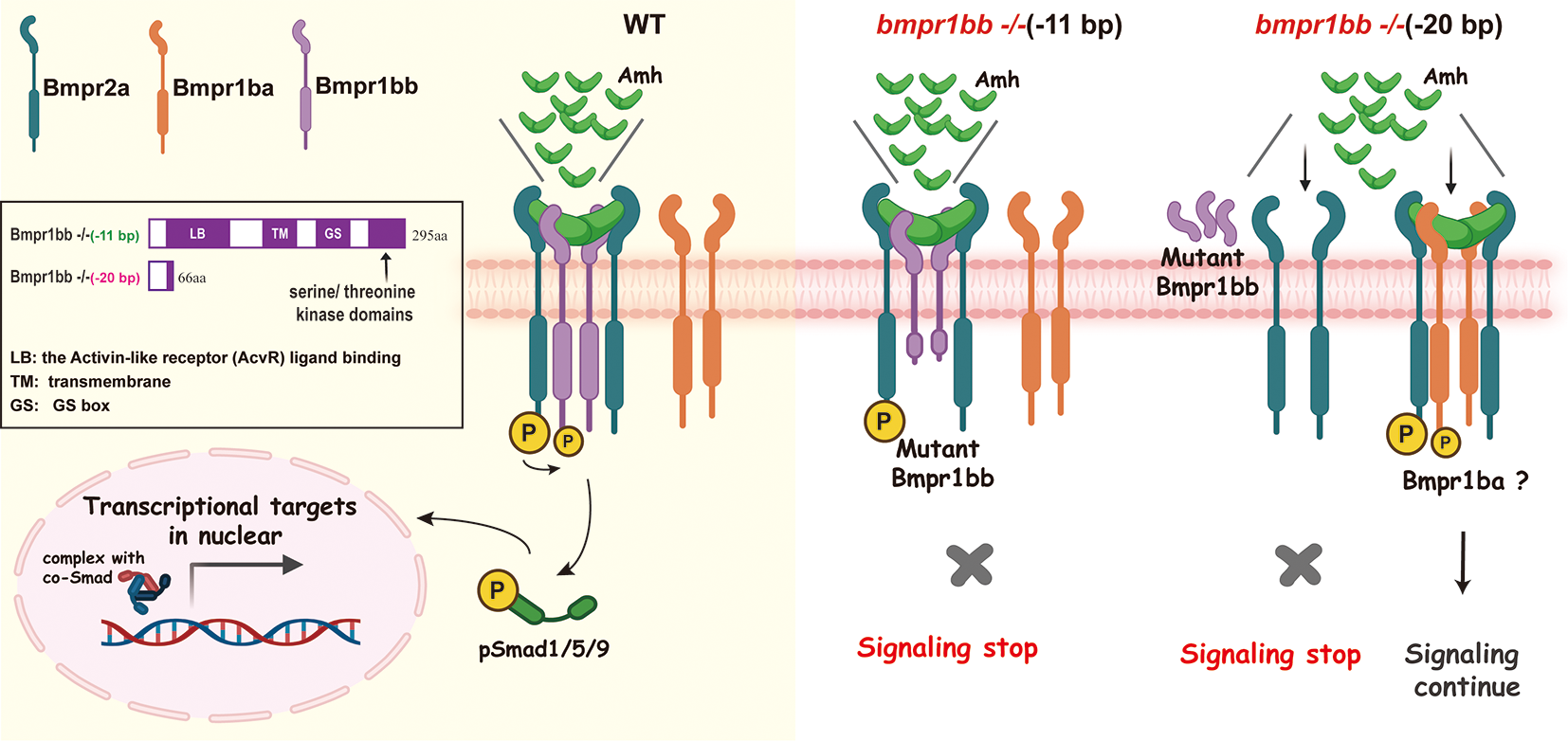

Supplement: S6 Fig — In WT, Amh signals through Bmpr2a and Bmpr1bb. The bmpr1bb (-11 bp) mutant retains the GS domain, allowing potential ligand binding. However, the mutant Bmpr1bb is unable to activate R-Smad and also inhibits other type I receptors, such as Bmpr1ba, from associating with Bmpr2a, thereby functioning as a dominant negative mutant. By comparison, the bmpr1bb (-20 bp) mutant lacks all functional domains, thereby allowing alternative type I receptors such as Bmpr1ba to couple with Bmpr2a in its absence. (TIF) [file pgen.1011958.s007.tif]

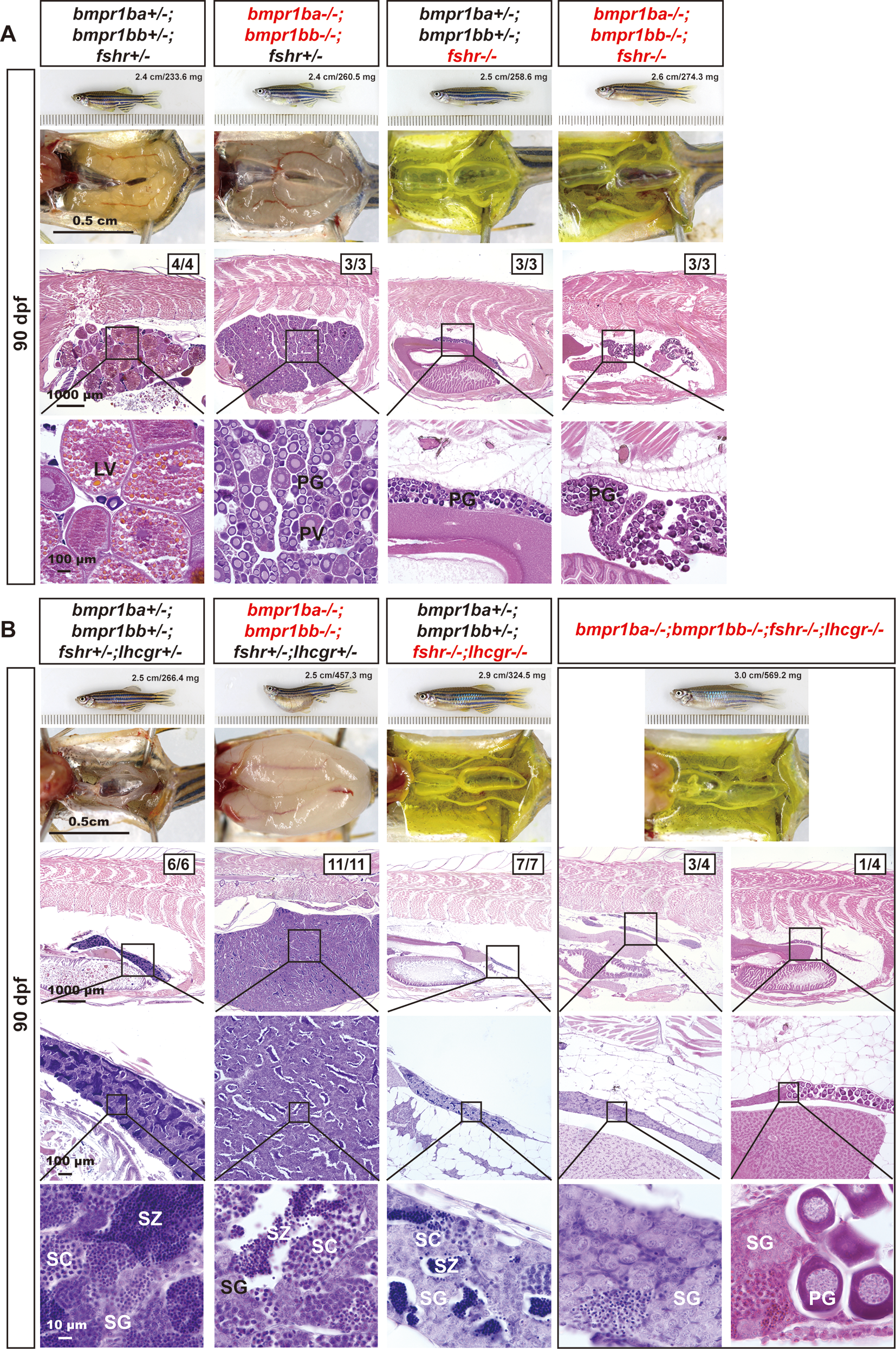

Supplement: S7 Fig — (A) Ovarian morphology and histology at 90 dpf in different genotypes. Double bmpr1b mutation (bmpr1ba-/-;bmpr1bb-/-) induced ovarian hypertrophy, characterized by the accumulation of early-stage follicles (PG and PV), while fshr mutation (bmpr1ba + /-;bmpr1bb + /-;fshr-/-) resulted in ovarian hypotrophy with only immature PG follicles. Triple mutation in both bmpr1b and fshr (bmpr1ba-/-;bmpr1bb-/-;fshr-/-) rescued the hypertrophic phenotype observed in the double mutants (bmpr1ba-/-;bmpr1bb-/-). (B) Testis morphology and histology at 90 dpf in different genotypes. Double bmpr1b mutation (bmpr1ba-/-;bmpr1bb-/-) showed hypertrophy and disrupted spermatogenesis. Double mutation of fshr and lhcgr (bmpr1ba + /-;bmpr1bb + /-;fshr-/-;lhcgr-/-) resulted in testicular hypotrophy with minimal production of SZ. The quadruple mutants (bmpr1ba-/-;bmpr1bb-/-;fshr-/-;lhcgr-/-) showed no testicular hypertrophy displayed by the double bmpr1b mutant (bmpr1ba-/-;bmpr1bb-/-). One out of four individuals displayed PG follicles in the testis. PG, primary growth; PV, pre-vitellogenic; LV, late vitellogenic; SG, spermatogonia; SC, spermatocytes; SZ, spermatozoa. (TIF) [file pgen.1011958.s008.tif]
