# Supplementary material for: Genetic and functional characterization of AMH Signaling in Zebrafish - Evidence for Roles of Amh-Bmpr2a-Bmpr1bb Pathway in Controlling Gonadal Homeostasis
Source: PLoS Genet. 2026 Mar 23;22(3):e1011958. doi: 10.1371/journal.pgen.1011958 (PMC13095126; doi:10.1371/journal.pgen.1011958)
Supplement: Attachment — Submitted filename: Response to Reviewers.docx [file pgen.1011958.s010.docx]

**Response to Reviewers**

**Reviewer #1**

It was known that zebrafish and other fish species lack Amhr2, the gene corresponding to Amh. Yue et al. investigated that bmp2a functions as the type 2 receptor and bmpr1bb as the type 1 receptor in AMH signaling. In addition to genetic analysis, they conducted detailed investigations, including demonstration that activation of SMAD in gonads by recombinant AMH requires Bmp2a. It provides solid evidence demonstrating which receptors function as AMH signaling receptors in zebrafish lacking Amhr, particularly regarding the type 2 receptor. Before publication, the reviewer requests some improvements, mainly additional explanation.

**Response:** We appreciate the Reviewer’s positive and encouraging comments on our study.

Major Comments

1. In Fig. 1E, the co-expression of type 1 receptors with bmp2a/2b is too low for 1ba and 1bb, making their relationship unclear. In Line 405, authors suggests bmpr1ba and 1bb are involved, but this figure lacks analysis. If extracting this data is feasible, please include it. If not, please explain the reason why bmpr1ba and 1bb were not examined.

**Response:** We appreciate the Reviewer pointing this out. We agree that the co-expression percentage (4.4% for *bmpr2a* and *bmpr1bb*) appears low in the scRNA-seq dataset. However, it is important to note that this dataset comes from 40-dpf ovaries. At this stage, the cell population mediating Amh-Bmpr2a-Bmpr1bb signaling is likely a small subpopulation of granulosa cells. Despite this, the dysfunction of this specific and critical subpopulation can lead to the dramatic phenotypes observed later in development.

We have revised the text to clarify that while the co-expression percentage is low, it represents a physiologically critical cell population, and our conclusion relies primarily on the functional genetic evidence (mutants) rather than expression levels alone. We did not include two paralogues of type I receptor Bmpr1a (*bmpr1aa* and *bmpr1ab*) in the analysis because it is beyond the scope of the current study. Another student in our laboratory has recently generated mutants for these two receptors, and she is now focusing on phenotype analysis.

*Changes to manuscript (Discussion section, Page 24):"**Although the co-expression was observed in a relatively small fraction of cells (~5.3%),* *this subpopulation likely represents a critical lineage of granulosa cells mediating Amh control of early folliculogenesis. The functional significance of this co-expression is supported by* *the strong genetic evidence, suggesting that the shared phenotypes observed in bmpr2a and bmpr1bb mutants may arise from dysfunction of this specific subpopulation of granulosa cells. Given the dynamic nature of folliculogenesis, it is plausible that the proportion of this granulosa cell subpopulation, characterized by bmpr2a and bmpr1bb co-expression, may change during follicle development."*

2. AMH administration experiments were only performed on type 2 receptors, making type 1 receptor analysis relatively weak. Despite such smaller amount of evidence, the author concluded that bmpr1bb is the main type 1 receptor in AMH signaling with similar intensity as Bmp2a. Please explain why the authors can conclude Bmp1bb is the coupling type 1 receptor for Bmp2a in AMH signaling.

**Response:** We thank the Reviewer for raising this point. We acknowledge that we did not perform the *in vitro* phosphorylation assay for the type 1 receptors as we did for type 2. This is largely because *bmpr1bb* mutant females exhibited a more severe phenotype than *amh* and *bmpr2a* mutants, as described in the manuscript. As a result, it is challenging to obtain sufficient follicle cells, most of which are derived from advanced vitellogenic follicles, for the in vitro experiment. Despite this shortcoming, our extensive genetic data provided solid and definitive evidence supporting the potential role of *bmpr1bb* in Amh-Bmpr2a signaling. In zebrafish genetics, phenotypic copying is a well-accepted approach for pathway identification. The *bmpr1bb* mutant phenocopies the *amh* and *bmpr2a* mutants on all aspects (gonadal hypertrophy, accumulation of early germ cells, rescue by disruption of gonadotropin signaling), whereas the *bmpr1ba* mutant is indistinguishable from the wild type. Furthermore, the double mutants (*bmpr1ba-/-;bmpr1bb-/-*) did not show a significantly different phenotype from the *bmpr1bb* single mutant, other than increased severity, suggesting *bmpr1bb* is the dominant player.

We have added a sentence to the Discussion to explicitly state that our conclusion regarding the type 1 receptor relies on the genetic data.

*Changes to manuscript (Discussion section, Page 25):* *"**Although we were unable to perform in vitro ligand–receptor activation assays for the type I receptors, primarily due to the limited availability of advanced follicles required for establishing follicle cell cultures, the in vivo genetic evidence is solid and compelling, supporting our proposal that Bmpr1bb, rather than Bmpr1ba, functions as the requisite type I receptor in this signaling pathway."*

**3.** Line 572 states, “These results suggest that the Amh-Bmpr2a-Bmpr1bb pathway may work by suppressing gonadotropin signaling.” However, couldn't this be explained even if the Amh system and FSH/LH actions were independent?

**Response:** This is an excellent question regarding epistasis. If the pathways were independent, we would expect that removing the gonadotropin receptors in an *amh* (or *bmpr2a/bmrp1bb*) mutant background would result in an additive phenotype or a partial rescue, rather than a complete reversion to the *fshr/lhcgr* mutant phenotype. The fact that the *fshr* loss-of-function phenotype (hypotrophy) completely overrides the *amh/bmpr2a/bmpr1bb* loss-of-function phenotype (hypertrophy) indicates that the hypertrophy caused by lack of Amh signaling is *dependent* on functional gonadotropin signaling. In genetic terms, this suggests that Amh signaling acts upstream to negatively regulate the sensitivity or output of the gonadotropin pathway.

We have clarified this point in the text to ensure the reader understands why we infer a direct suppression mechanism.

*Changes to manuscript (Discussion section, Page 26):* *"**If the Amh and gonadotropin pathways operated independently, one might expect an additive phenotype or partial rescue in the combined mutants. However, the complete abrogation of gonadal hypertrophy in the absence of gonadotropin receptors demonstrates that the hypertrophic phenotype is strictly dependent on gonadotropin signaling, supporting a model where Amh acts as a 'brake' on gonadotropin responsiveness."*

**Reviewer #2**

In this manuscript, Ge and colleagues address an unresolved and important question regarding the hormone AMH in the zebrafish reproductive... Overall, the manuscript presents a robust amount of data, and the authors’ conclusions are convincing. I have a few questions/suggestions that may further strengthen this strong and comprehensive study.

**Response**: We appreciate the Reviewer’s positive and encouraging comments on our study.

1. On page 14 (first paragraph), the authors report the co-expression of bmpr2a with bmpr2b, bmpr1ba, and bmpr1bb. According to the expression data provided, bmpr2a is co-expressed with bmpr2b in ~7% of granulosa cells, with bmpr1bb in ~4.3%, and with bmpr1ba in ~6.4%. The paragraph as written seems to imply that these co-expression patterns support the involvement of bmpr1ba and bmpr1bb, but not bmpr2b. However, the co-expression data alone do not exclude a possible role for bmpr2b. While the subsequent in vitro biochemical data do suggest that bmpr2b may indeed not be required, this specific paragraph should be reworded to avoid overinterpreting the expression patterns.

**Response:** We agree with the reviewer. Relying solely on expression overlap to exclude *bmpr2b* is premature at that point in the manuscript. We have modified the text to soften this claim, stating that the expression patterns are *consistent* with our hypothesis, but that functional testing (which we present later) is required to rule out *bmpr2b*.

*Changes to manuscript (Results section, Page 15):* *"**Finally, the abundance of bmpr2b-expressing cells was much lower than that expressing bmpr2a... indicating that Bmpr2b signaling at this stage of follicle development may involve other BMP type I receptors, such as bmpr1aa and bmpr1ab (Fig. 1E). Although the expression data are informative, we cannot completely rule out a role for Bmpr2b in Amh signaling without functional verification (see below)."*

2. The description of the bmpr1bb -11 mutant on page 15 (last paragraph) and page 16 (first paragraph) raises an important interpretive issue. The -11 mutant fish have more severe phenotypes compared to -20. The authors hypothesize that the -11 allele produces a truncated receptor that may act in dominant-negative manner activity... I suggest the authors more explicitly discuss how the dominant nature of the Δ11 allele may affect later analyses and whether some phenotypes might arise from non-physiological receptor activity.

**Response:** We appreciate this insightful comment. The difference between the -11 (truncated/potential dominant-negative) and -20 (likely null) alleles is indeed obvious, and we discussed this issue in the manuscript and illustrated the idea in Figure S6. There are two explanations for the observed discrepancy between the two mutants. First, as speculated in the paper, the truncated form (-11) may function as a potential dominant negative by sequestering shared type II receptors or ligands, thereby inhibiting other BMP pathways beyond just Amh signaling. Second, the complete absence of Bmpr1bb in the *bmpr1bb* (–20) mutant may force Bmpr2a to recruit alternative TGF‑β family type I receptors, thereby partially restoring Smad1/5/9 signaling. This will be an interesting issue to explore in future studies. We have added a dedicated passage in the Discussion to address the specific nature of the -11 allele and how it might exaggerate the phenotype compared to a simple loss-of-function.

*Changes to manuscript (Results section, Page 17):* *"**There are two explanations for the observed discrepancy between the two mutants. First, the truncated form (-11) may function as a potential dominant negative mutant by sequestering shared type II receptors or ligands (Fig. S6), thereby inhibiting other BMP pathways beyond just Amh signaling. Second, the complete absence of Bmpr1bb in the bmpr1bb (–20) mutant may force Bmpr2a to recruit alternative and physiologically* *irrelevant TGF‑β family type I receptors, thereby partially restoring Smad1/5/9 signaling. The exact mechanism is unknown, and it will be an interesting issue to explore in future studies."*

3. This is optional. The manuscript contains a remarkable and huge dataset... The MS is very long, and in some places- particularly in the Discussion section, there is redundancy with earlier sections. I recommend streamlining the manuscript by reducing repetitive descriptions and tightening the narrative.

**Response:** We appreciate the Reviewer’s suggestion. We have substantially reduced the length of the Discussion section by removing several paragraphs that merely restated the Results without adding interpretive value. This has improved the flow and reduced the overall word count.

**Reviewer #3**

This is a comprehensive study investigating the signalling pathway involved in AMH-regulated gonadal development... However, several issues need to be addressed.

**Response:** We appreciate the Reviewer’s positive and encouraging comments on our study.

**1.** Fig. 1C shows that amh is expressed at the highest level in PV and the level declines with follicle growth and reaches the lowest level in FG. However, both bmpr2a and bmpr1bb mRNA reach the highest level in FG. The discrepancy in expression patterns between amh and its receptors should be discussed. Are their other types for receptors for Amh? Do bmpr2a and bmpr1bb mediate the function of other TGF-beta family members?

**Response:** We thank the Reviewer for raising this interesting issue. The high expression of *bmpr2a* and *bmpr1bb* in late stages (FG), where *amh* is low, strongly suggests that these receptors are promiscuous and likely mediate signaling for other TGF-β superfamily ligands, especially other members of BMP family during later folliculogenesis. Our previous study has demonstrated that recombinant Bmpr2a and Bmpr2b both respond to recombinant zebrafish BMP4 (Li and Ge 2011 Biol. Reprod. 85, 977–986). We discussed this issue in the manuscript. To further address this receptor promiscuity, we have revised and expanded the part in Discussion as follows.

*Changes to manuscript (Discussion section, Page 24):* *"**The temporal expression mismatch between Amh and bmpr2a suggests that Bmpr2a is not dedicated solely to Amh. It is highly possible that Bmpr2a together with Bmpr2b also mediate signaling for other TGF-β superfamily ligands, especially other members of BMP family, during later stages of vitellogenic growth. This idea is supported by our early study demonstrating that recombinant zebrafish Bmpr2a and Bmpr2b could both respond to recombinant zebrafish BMP4 (43)."*

2. Based on Fig. 1E (scRNA dataset, only a very small fraction (4.3%) of cells express bmpr2a+bmpr1bb, why do you think bmpr1bb KO has such dramatic effects?

**Response:** As described and discussed in the manuscript, the scRNA data revealed high level of heterogeneity among granulosa cells. As responded to Reviewer #1, we believe that although the population of *bmpr2a*+*bmpr1bb*-expressing cells is small, this subset of cells represents a critical "gatekeeper" lineage of granulosa cells responsible for the recruitment of follicles into the secondary growth phase. Even if the population is small, its regulatory role is pivotal; failure in this specific subset leads to the dysregulation of the entire follicle, resulting in the accumulation of PG follicles. It is important to note that the scRNA-seq data were obtained from 40-dpf follicles, a stage immediately following gonadal differentiation. The proportion of *bmpr2a*+*bmpr1bb*-expressing cells may increase during later stages of folliculogenesis given the dynamic nature of the process. We have clarified this in the Discussion.

3. In the Materials and Methods, the authors state that one-way ANOVA was used for data analyses. However, Fig. 2B and Fig 3B should be analyzed by two-way ANOVA, not one-way.

**Response:** We thank the reviewer for this insightful comment regarding our statistical approach. We agree that the experimental design involves two factors: genotype and time. However, our primary biological question was specifically focused on phenotypic differences between genotypes at specific developmental stages or age, rather than the progression of growth over time.

Since gonadal growth (GSI) increases significantly and naturally over time, comparing time points against each other (*e.g*., comparing 80 dpf vs. 150 dpf) does not yield biologically meaningful insights for our study's objectives. In addition, because the variance due to age (time) is very large, including it in the analysis using two-way ANOVA may obscure subtle differences between genotypes. That is the reason we chose one-way ANOVAs at each time point (80, 100, and 150 dpf) to maximize the power to detect genotypic differences. We have clarified this rationale in the figure legends to avoid confusion.

*Changes to manuscript (figure legends of Fig. 2 and 3):* *"* *Statistical differences were analyzed using one-way ANOVA performed independently at each time point (80, 100, and 150 dpf). Different letters indicate significant differences (P < 0.05) between genotypes within the respective age group."*

4. Fig. 5. For all western blots with pSmad1/5/9, total Smad1/5/9 must also be measured to confirm the activation, rather than a stimulation of Smad expression.

**Response:** We agree with the Reviewer that normalizing phosphorylated proteins to their total protein counterparts is a common approach for confirming pathway activation. We would have preferred to include total Smad1/5/9 data; however, we faced a technical limitation commonly encountered in the zebrafish model system. Most commercially available antibodies are raised against mammalian immunogens. In our experience, it is not uncommon that the antibodies for total proteins exhibit poor cross-reactivity and low specificity when applied to zebrafish samples, often resulting in multiple non-specific bands that make quantification unreliable. In contrast, the antibodies against phosphorylated proteins like pSmad1/5/9 target phosphorylation motifs, which are usually highly conserved across species, allowing for high specificity and clean detection in zebrafish tissues. For this reason, we chose to normalize our data using two well-established housekeeping proteins, beta-actin and GAPDH. These proteins demonstrated stable expression across our samples and served as reliable internal loading controls. Furthermore, our study focuses on the immediate cellular responsiveness to Amh in cultured follicle cells (30 min). Since the signaling response (phosphorylation) occurs rapidly, it is unlikely that the observed differences are driven by *de novo* synthesis of total Smad proteins within this short timeframe. Therefore, we are confident that the changes observed in the pSmad1/5/9 levels normalized to housekeeping proteins accurately reflect the activation status of the pathway.

5. The results from the cross between bmpr mutants and fshr/lhcgr mutants are somewhat difficult to follow. Please include a diagram to show the different lines generated and key finding.

**Response:** We understand that the complex genetic crosses can sometimes be difficult to follow. As suggested by the Reviewer, we have added a new figure (Fig. 11) that provides a schematic representation of the crossing schemes and the resulting phenotypes for different combinations of mutants.

6. Include a summary diagram to illustrate how amh exerts its functions via bmprs and the interaction between this pathway and gonadotropin signaling pathways.

**Response:** As responded to point 5 above, we have added a summary model in new Figure 11 that illustrates the Amh-Bmpr2a-Bmpr1bb signaling cascade and its inhibitory effect on Fshr/Lhcgr signaling. This figure visually summarizes the core findings of our study: Amh binds Bmpr2a/Bmpr1bb to phosphorylate Smad1/5/9, which in turn dampens the sensitivity of the gonads to gonadotropins FSH/LH. The loss of such gatekeeping mechanism leads to over-stimulation of the gonads by gonadotropins, resulting in gonadal hypertrophy.
